# Quantitative Trait Locus Analysis and Identification of Candidate Genes Affecting Seed Size and Shape in an Interspecific Backcross Inbred Line Population of *Gossypium hirsutum* × *Gossypium barbadense*

**DOI:** 10.3389/fpls.2022.837984

**Published:** 2022-03-22

**Authors:** Luyao Wu, Bing Jia, Wenfeng Pei, Li Wang, Jianjiang Ma, Man Wu, Jikun Song, Shuxian Yang, Yue Xin, Li Huang, Pan Feng, Jinfa Zhang, Jiwen Yu

**Affiliations:** ^1^Zhengzhou Research Base, State Key Laboratory of Cotton Biology, School of Agricultural Sciences, Zhengzhou University, Zhengzhou, China; ^2^State Key Laboratory of Cotton Biology, Key Laboratory of Cotton Genetic Improvement, Ministry of Agriculture, Institute of Cotton Research of Chinese Academy of Agricultural Sciences, Anyang, China; ^3^Department of Plant and Environmental Sciences, New Mexico State University, Las Cruces, NM, United States

**Keywords:** *Gossypium hirsutum*, *Gossypium barbadense*, backcross inbred lines, seed size and shape, quantitative trait locus, candidate genes

## Abstract

Seed size and shape are key agronomic traits affecting seedcotton yield and seed quality in cotton (*Gossypium* spp.). However, the genetic mechanisms that regulate the seed physical traits in cotton are largely unknown. In this study, an interspecific backcross inbred line (BIL) population of 250 BC_1_F_7_ lines, derived from the recurrent parent Upland CRI36 (*Gossypium hirsutum*) and Hai7124 (*Gossypium barbadense*), was used to investigate the genetic basis of cotton seed physical traits via quantitative trait locus (QTL) mapping and candidate gene identification. The BILs were tested in five environments, measuring eight seed size and shape-related traits, including 100-kernel weight, kernel length width and their ratio, kernel area, kernel girth, kernel diameter, and kernel roundness. Based on 7,709 single nucleotide polymorphic (SNP) markers, a total of 49 QTLs were detected and each explained 2.91–35.01% of the phenotypic variation, including nine stable QTLs mapped in at least three environments. Based on pathway enrichment, gene annotation, genome sequence, and expression analysis, five genes encoding starch synthase 4, transcription factor PIF7 and MYC4, ubiquitin-conjugating enzyme E27, and THO complex subunit 4A were identified as candidate genes that might be associated with seed size and shape. Our research provides valuable information to improve seed physical traits in cotton breeding.

## Introduction

Cotton (*Gossypium* spp.) is an important cash crop, grown for monetary profit from the fiber, feed, and cooking oil. Currently, research on cotton predominantly focuses on fiber yield and quality, with relatively few studies on seed quality (Deng et al., 2019; Chen et al., 2020; Liu H. et al., 2020). Seed quality is one of the most important factors considered in cotton stand establishment procedures (Sawan and Dello Ioio, 2016). Although cotton production has seen technological advances, the lack of quality cottonseed may be perceived as a pertinent issue. Good quality seeds of improved cultivars comprise one of the key inputs for attaining high cotton yield with increased economic benefits (Atique-ur-Rehman et al., 2020).

Seed size is a widely accepted measure of seed quality, and multiple earlier studies have shown that large seeds have high capacities for seedling survival, growth, and establishment (Lehtilä and Ehrlén, 2005). Compared to small-seed cultivars, large-seed cultivars exhibit increased fiber length, strength, and decreased micronaire (Main et al., 2013). Compared to small-size and mixed-size seeds, large-size and medium-size seeds achieved increased germination potential, germination rate, seed fullness, dry matter weight per plant, root-to-shoot ratio, leaf emergence rate, and leaf area (Liu et al., 1997). Seed size is the primary factor considered during harvesting and processing (Atique-ur-Rehman et al., 2020). Individual plant seed mass, in addition to total oil and protein energy content, predicts early seedling vigor (Snider et al., 2016). Additionally, oil content is largely affected by seed size (Pahlavani et al., 2008).

Quantitative trait locus (QTL) mapping uses molecular markers, based on genetic linkage maps, to determine the position of DNA segments or genes that control quantitative traits (Powder, 2020). Studies employing QTL mapping in cotton have predominantly focused on the QTL location of fiber traits (Said et al., 2013, 2015), with the related molecular mechanisms gradually revealed over time (Tian and Zhang, 2021). QTL mapping for seed quality tends to focus on oil content (Yu et al., 2012; Liu et al., 2013; Liu C. et al., 2020), protein content (Yu et al., 2012; Liu et al., 2013) and other aspects (Liu et al., 2008). Few studies have described QTL locations for seed physical traits including size and shape (Wang et al., 2019).

It is known that seed size and shape is a complex quantitative trait that is controlled by multiple genes in crops. In other crops, more QTL mapping and research on seed size and shape-related traits have been conducted, such as peanuts (Zhang et al., 2019), soybeans (Hina et al., 2020), and rice (Ying et al., 2018). Using specific site amplified fragments (SLAF) sequencing- based 7,033 single nucleotide polymorphic markers (SNPs) to construct a genetic map in a recombinant inbred line (RIL) population of 180 Upland cotton lines, Wang et al. (2019) identified 32 QTLs for four traits related to seed size, i.e., hundred seed weight (HSW), hundred kernel weight (HKW), ten kernel length (TKL), and ten kernel width (TKW). However, molecular and genomic studies on cottonseed physical traits are currently lacking. It is known that cottonseed size is associated with seedling vigor, and oil and protein content. Seed size and seed shape also affect the seed surface area, which in turn could affect the number of fiber initials and eventually lint fibers. The number of lint fibers and their length and fineness are important determinants of lint percentage, a lint yield component trait. Therefore, QTL mapping of cotton seed size and shape-related traits is of great significance for revealing the molecular mechanism of cotton seed development and improving cotton yield, seed, and fiber quality.

In a previous study, a backcross inbred line (BIL) population containing 250 BC_1_F_7_ lines, derived from an interspecific cross between recurrent parent *Gossypium hirsutum* L. CRI36, and *Gossypium barbadense* L. Hai7124, was developed and SLAF sequencing was used for SNP typing (Ma et al., 2019). The objectives of this study were to perform a QTL analysis for seed size and shape in this BIL population. Eight traits related to seed size and shape were assessed: 100-kernel weight, kernel length, kernel width, kernel length to width ratio, kernel area, kernel girth, kernel diameter, and kernel roundness. To lay a theoretical foundation for improving the quality of cotton seeds and furthering research on related genetic mechanisms, we also analyzed candidate genes for stable QTL intervals.

## Materials and Methods

### Plant Material and Generation of Phenotypic Data

An interspecific BIL population containing 250 BC_1_F_7_ lines was developed from a cross between *G. hirsutum* CRI36 (as the recurrent parent) and *G. barbadense* Hai7124. Ma et al. (2019) described the development details of the BILs and created a genetic linkage map composed of 7,709 SNP markers. The parents and 250 BILs were planted in five environments according to a randomized complete block design with two replications in each environment. Three field tests were conducted in the experimental farm, CRI, CAAS, Anyang (Henan Province, 36.06°N, 114.49°E) with one test in 2016 and two tests (one in the south farm and another in the east farm) in 2017. Two field tests were conducted in Shihezi (Xinjiang Uygur Autonomous Region, 44.44°N, 85.68°E) in 2016 and 2017. In each test, cotton seeds were hill-sown by hand and covered with plastic mulch applied directly by a machine in April each year. In Anyang, approximately 16 plants per 4-m-long row were retained, and the row spacing was 0.80 m. In Xinjiang, where a high-density seeding rate was used, approximately 44 plants per 5-m-long row were retained, and the row spacing was 0.38 m. Crop management practices followed the recommendations of local cotton production. The use of the two cotton production systems (i.e., normal and high plant density) allowed detection of consistent QTLs for the seed physical traits between the two production systems. The average best linear unbiased prediction (BLUP) of the five environments was also calculated and used for QTL mapping. We conducted SLAF sequencing with the *G. hirsutum* genetic standard TM-1 as a reference genome (Zhang et al., 2015; Hu et al., 2019) to genotype the BILs.

Twenty opened bolls were manually harvested at crop maturity. After ginning and acid-delinting of the cottonseed, a Wanshen SC-G Automatic Seed Test Analyzer was used to determine the properties of cotton kernels: the 100-kernel weight (HKW, g), kernel length (KL, mm), kernel width (KW, mm), kernel length to width ratio (KLW), kernel area (KA, mm^1^), kernel girth (KG, mm), kernel diameter (KD, mm), and kernel roundness (KR, mm). Analysis of variance, the frequency distribution and correlation coefficients among these traits were analyzed using SPSS (version 20.0; SPSS, Chicago, IL, United States). The lme4 package in R was used to estimate the BLUP value of the five environments, enabling its use in correlation analysis of the eight traits (Poland et al., 2011).

### Quantitative Trait Locus Analysis

Inclusive Composite Interval Mapping (ICIM) in the IciMapping4.2 software was used to perform QTL analyses for each seed physical trait (Meng et al., 2015). The threshold of logarithm of the odds (LOD) value was set using 1,000 permutation tests, and the detection step was set to 1 cM. Positive additive effects indicated favorable alleles derived from CRI36, while negative additive effects indicated favorable alleles from Hai7124. A QTL identified in three or more environments were considered a stable QTL (Shang et al., 2015). The naming method of QTLs followed a previous report (Gu et al., 2020). MapChart (version 2.2) was used for constructing linkage maps for mapped SNPs with QTL intervals indicated.

### Candidate Gene Identification and Annotation

The physical interval of each stable QTL was determined using Basic Local Alignment Search Tool (BLAST) (Hu et al., 2019). Potential candidate genes related to seed size and shape traits were determined based on Gene Ontology (GO) enrichment and Kyoto Encyclopedia of Genes and Genomes (KEGG) analyses. GO and KEGG analyses were performed using OmicShare tools^2^ (Li et al., 2020). These genes were identified using CottonFGD.^2^ The functions of the identified genes were determined through gene annotation. The *Arabidopsis thaliana* homologous genes and gene function annotations of candidate genes were determined using the TM-1 genome. Based on CRI36 and Hai7124 resequencing (30×) results, candidate genes were further screened by SNP variation between the two parents. Polymorphic loci with missing or heterozygous genotypes, as well as polymorphic loci without polymorphism between parents, were filtered out. The remaining SNPs and indels were considered as effective polymorphic loci. SnpEff 4.2 software was used to predict the function of these effective polymorphic loci based on the published cotton genome sequence annotations (Cingolani et al., 2012).

Because the gene expression data for the Upland cotton parent CRI36 were not available, the expression levels of candidate genes from the sequenced TM-1 were used as a proxy to compare with these from another parent- Hai7124, both of which were based on existing RNA sequencing (RNA-seq) data (National Genomics Data Center: accession number: PRJNA490626)^3^ (Hu et al., 2019). Candidate genes that were poorly expressed in cotton ovules (FPKM < 2) were discarded. Screening for genes that were specifically expressed in ovules or whose expression levels were significantly different in TM-1 and Hai7124 ovules, was performed. The fold change in candidate gene expression was set to 2 as the threshold for significant differential expression between TM-1 and Hai7124 in embryos (0, 1, 3, 5, 10, and 20 days post anthesis, DPA).

## Results

### Phenotypic Performance in the Backcross Inbred Line Population of *Gossypium hirsutum* CRI36 and *Gossypium barbadense* Hai7124 in Five Environments

The traits of 100-kernel weight (HKW), kernel length (KL), kernel width (KW), kernel length to width ratio (KLW), kernel area (KA), kernel girth (KG), kernel diameter (KD), and kernel roundness (KR) were used to evaluate the seed size and shape of the parents, *G. hirsutum* CRI36 and *G. barbadense* Hai7124, and their interspecific BIL population in five environments. The traits of KLW, KL, KR, and KW significantly differed between the two parental lines of different species; and *G. barbadense* Hai7124 seeds were shorter and more rounded than the seeds of *G. hirsutum* CRI36. HKW, KA, KG, and KD did not significantly differ between the two parents (Table 1). However, analysis of variance (ANOVA) detected significant genetic variations for all the seed physical traits in the BIL population including these traits for which the two parents did not differ (Supplementary Table 1). The results indicate that different genes controlling the same traits with similar values between the two species, resulting in transgressive segreation.

**TABLE 1:** Comparison of the seed size and shape-related traits between two parents *Gossyium hirsutum* CRI36 and *G. barbadense* Hai7124.

	HKW	KA	KG	KLW	KL	KW	KD	KR
CRI36	7.28 ± 1.42	25.62 ± 3.32	21.61 ± 1.45	2.12 ± 0.06	8.45 ± 0.52	3.99 ± 0.26	5.69 ± 0.36	0.47 ± 0.01
Hai7124	7.5 ± 0.82	24.82 ± 1.59	20.28 ± 0.54	1.78 ± 0.08**	7.62 ± 0.17**	4.31 ± 0.18*	5.61 ± 0.18	0.57 ± 0.02**

* and ** mean significant at P < 0.05 and P < 0.01, respectively. Traits in the top horizontal row: 100-kernel weight (HKW, g), kernel area (KA, mm^2^), kernel girth (KG, mm), kernel length to width ratio (KLW), kernel length (KL, mm), kernel width (KW, mm), kernel diameter (KD, mm), and kernel roundness (KR, mm).

**FIGURE 1:**
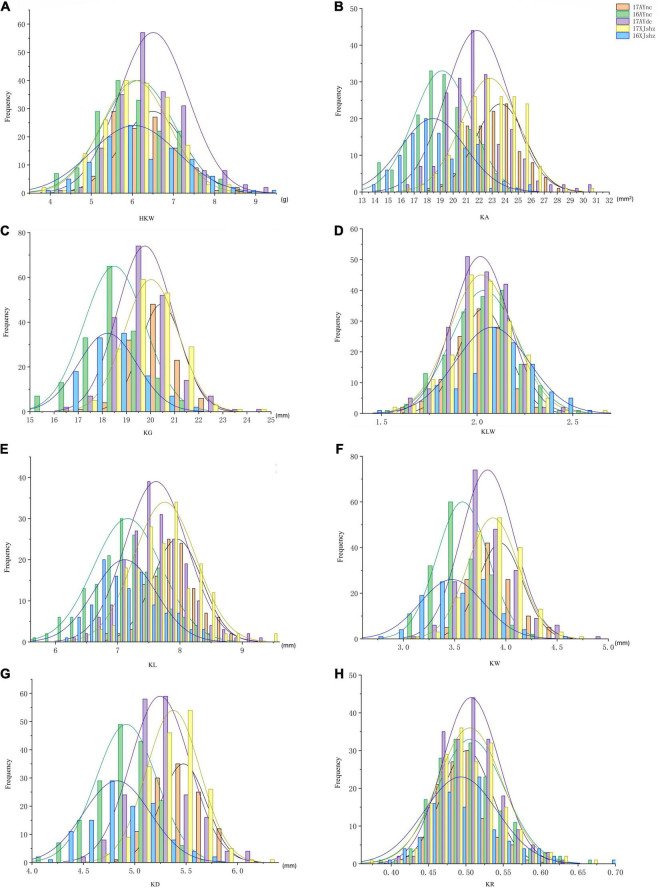
Frequency map of 8 traits in different environments of 250 BILs. Different colors represent different environments. **(A)** HKW. **(B)** KA. **(C)** KG. **(D)** KLW. **(E)** KL. **(F)** KW. **(G)** KD. **(H)** KR. See the footnote in Table 1 for explanations of the abbreviations.

The results of the descriptive statistics of phenotypic data for all traits in the five environments (except BLUP) of BIL are shown in Supplementary Table 2. Broad-sense heritability estimates were 0.74–0.86, indicating that all traits were mainly affected by the genotype. Both the skewness and kurtosis values of the eight traits in the five environments were < 1.0 except for a few cases, indicating that none of the traits deviated significantly from a normal distribution (Figures 1A–H). We further calculated correlation coefficients among the eight seed size and shape-related traits in the BIL population; there were 24 significant correlations between the eight traits (Table 2). Among them, HKW, KL, KW, KA, KG, and KD showed a positive correlation. KLW was significantly and negatively correlated with HKW, KW, and KR, and KR was significantly negatively correlated with KG, KLW, and KL. The same cottonseed physical trait was significantly correlated among different environments, suggesting the environmental stability of these traits. Taking HKW as an example, the correlation between various environments was analyzed, and it was found that there was a significant positive correlation among all environments (Supplementary Table 3).

**TABLE 2:** Correlation coefficients among cotton seed size and shape-related traits in the BIL population.

Trait	HKW	KA	KG	KLW	KL	KW	KD	KR
**HKW**	1							
**KA**	0.949**	1						
**KG**	0.794**	0.928**	1					
**KLW**	−0.240**	0.006	0.393**	1				
**KL**	0.572**	0.780**	0.961**	0.617**	1			
**KW**	0.889**	0.798**	0.505**	−0.586**	0.265**	1		
**KD**	0.925**	0.999**	0.911**	0.001	0.780**	0.801**	1	
**KR**	0.253**	0.009	−0.376**	−0.989**	−0.603**	0.601**	0.013	1

** means significant at P < 0.01. Traits column refers to the eight seed size and shape characteristics defined in Table 1.

### Quantitative Trait Locus Analysis of Cottonseed Physical Traits

Based on the high-density genetic map and phenotypic data, a total of 49 QTLs were detected on 14 chromosomes, including 28 and 21 QTLs in the A and D subgenomes, respectively. These included five QTLs for HKW, five for KL, five for KW, five for KLW, eight for KA, two for KG, twelve for KD, and seven for KR (Supplementary Figure 1). An LOD value of 3.64–15.6 was obtained for the QTLs, with 2.91–35.01% phenotypic variation explained (PVE) by each QTL (Supplementary Table 4). The PVE of QTLs for HKW, KL, KW, KLW, KA, KG, KD and KR ranged from 5.38 to 35.01%, 5.41 to 35.01%, 13.70 to 34.10%, 6.82 to 22.80%, 5.68 to 25.10%, 8.18 to 12.91%, 2.91 to 25.28%, and 5.59 to 20.84%, respectively. A total of nine QTLs were consistently detected in at least three environments, namely *qHKW-D03-1*, *qKW-D03-1*, *qKLW-D03-1*, *qKLW-D12-1*, *qKA-D03-1*, *qKG-D03-1*, *qKD-D03-1*, *qKR-D03-1*, and *qKR-D12-1* (Table 3). Among these nine stable QTLs, the additive effect of *qKLW-D03-1* and *qKLW-D12-1* came from CRI36, while the others came from the male parent Hai7124.

**TABLE 3:** Stable quantitative trait loci (QTLs) for cottonseed physical traits identified in five environments and BLUP.

Trait	QTL	Env	Position (cM)	Flanking markers	LOD	*R*^2^(%)	Add
**HKW**	***qHKW-D03-1***	16AYnc	9	Marker149953 Marker150605	10.02	17.42	-0.53
		17XJshz	17	Marker150834 Marker150795	9.53	22.75	–0.48
		17AYdc	18	Marker150867 Marker150998	10.03	21.72	–0.50
		16XJshz	18	Marker150867 Marker150998	15.60	35.01	–0.79
		BLUP	18	Marker150867 Marker150998	14.41	22.56	–0.39
**KW**	***qKW-D03-1***	16XJshz	16	Marker151011 Marker151014	15.23	34.10	–0.21
		17XJshz	17	Marker150834 Marker150795	7.20	13.81	–0.12
		17AYdc	18	Marker150867 Marker150998	5.60	13.70	–0.12
		BLUP	18	Marker150867 Marker150998	14.51	21.49	–0.10
**KLW**	***qKLW-D03-1***	16XJshz	16	Marker151011 Marker151014	5.84	16.21	0.08
		17AYnc	22	Marker151179 Marker151250	4.25	11.31	0.06
		BLUP	21	Marker151072 Marker151143	5.32	9.05	0.04
	***qKLW-D12-1***	17XJshz	20	Marker195389 Marker195415	4.86	10.48	0.07
		16XJshz	20	Marker195389 Marker195415	7.57	22.80	0.11
		BLUP	20	Marker195389 Marker195415	4.34	7.42	0.04
**KA**	***qKA-D03-1***	17AYdc	5	Marker150850 Marker149780	5.62	11.64	–1.29
		16AYnc	10	Marker149953 Marker150605	8.33	12.23	–1.18
		BLUP	10	Marker149953 Marker150605	10.09	16.88	–0.84
		17XJshz	17	Marker150834 Marker150795	7.03	16.75	–1.13
		16XJshz	18	Marker150867 Marker150998	12.30	25.10	–1.60
**KG**	***qKG-D03-1***	17AYdc	5	Marker150850 Marker149780	4.22	8.18	–0.50
		16XJshz	10	Marker149953 Marker150605	4.93	12.91	–0.57
		BLUP	8	Marker149953 Marker150605	4.94	9.56	–0.34
**KD**	***qKD-D03-1***	17AYdc	5	Marker150850 Marker149780	5.42	11.35	–0.15
		16AYnc	10	Marker149953 Marker150605	8.27	12.27	–0.15
		BLUP	10	Marker149953 Marker150605	11.21	9.61	–0.09
		17XJshz	17	Marker150834 Marker150795	6.92	16.51	–0.13
		16XJshz	18	Marker150867 Marker150998	12.24	25.28	–0.21
**KR**	***qKR-D03-1***	16XJshz	16	Marker151011 Marker151014	9.41	20.84	–0.03
		16AYnc	18	Marker150867 Marker150998	4.27	11.16	–0.02
		BLUP	21	Marker151072 Marker151143	6.56	10.10	–0.01
		17AYnc	22	Marker151179 Marker151250	4.72	12.22	–0.02
	***qKR-D12-1***	16XJshz	19	Marker195381 Marker195387	7.97	16.43	–0.03
		17XJshz	20	Marker195389 Marker195415	5.67	12.02	–0.02
		BLUP	20	Marker195389 Marker195415	3.68	5.59	–0.01

*Traits column refers to the eight seed size and shape characteristics defined in Table 1. Env column denotes the five environments and BLUP. Logarithm of odds (LOD) values, R^2^(%) – phenotypic variation explained by the QTL, and Add – additive effect, columns refer to statistical test values.*

The QTLs identified for all traits were not randomly distributed across chromosomes or chromosomal regions, some of which were closed linked in clusters. The QTLs for the same or different traits that shared an overlapping confidence interval or located in an adjacent region were estimated as the presence of a cluster (Said et al., 2013). A total of seven QTL clusters were identified in this study (Supplementary Table 5), among which four and three clusters were observed in the At and Dt subgenomes, respectively. These clusters were distributed on seven chromosomes, among which one cluster was located on each of A04, A07, A08, A13, D03, D09, and D12. The D03 QTL cluster contained the largest number of QTLs (7), followed by *qClu-D09-1* (5).

### Prediction of Candidate Genes in Stable Quantitative Trait Locus

**FIGURE 2:**
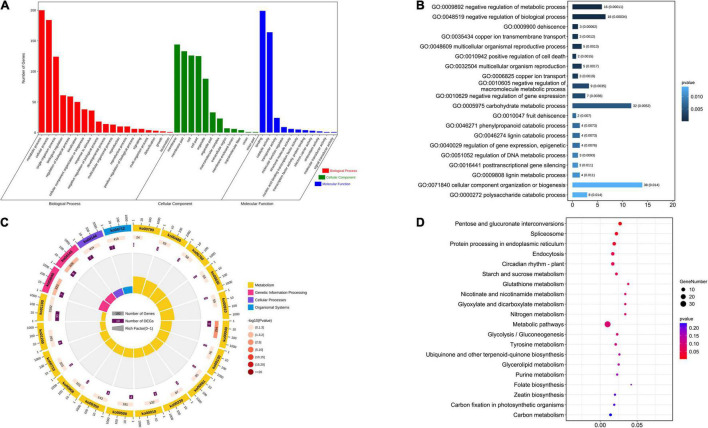
Analysis of the GO enrichment and KEGG of stable QTLs related to seed size and shape. **(A)** Analysis of the GO enrichment of stable QTLs. **(B)** Top 20 GO terms enrichment in the molecular function category. **(C)** Analysis of the KEGG of stable QTLs. **(D)** Top 20 of KEGG enrichment.

There were 641 candidate genes within the nine stable QTL regions. First, GO enrichment and KEGG analyses were performed on these candidate genes. In the GO analysis results (Figures 2A,B and Supplementary Table 6), the number of genes related to the metabolic process of the biological process category was 200. The number of genes identified as being related to cellular processes in the biological process category was 184, and the number of genes identified as being related to the combination of molecular functional categories was 199. Among the top 20 GO enrichment results, cellular component organization, or biogenesis, and carbohydrate metabolic process had the most enriched genes. In the KEGG analysis results (Figures 2C,D and Supplementary Table 7), the number of genes enriched in the metabolic pathway was 33, and 13 genes were enriched in the biosynthesis of secondary metabolites. In the top 20 KEGG pathways, several genes from the spliceosome (Jiang et al., 2011), protein processing in the endoplasmic reticulum (Yi et al., 2021), starch and sucrose metabolism (Yin et al., 2020), and purine metabolism (Qi and Xiong, 2013) were found to be related to seed size. In addition, in the remaining KEGG pathways, ABC transporters are also related to seed size (Do et al., 2018). Plant hormone signal transduction (Li et al., 2011; Liu et al., 2015; Ji et al., 2019), mitogen-activated protein kinase (MAPK) signaling (Li and Li, 2015) and ubiquitin-mediated proteolysis (Li et al., 2008; Xia et al., 2013) pathways have been studied extensively in the context of seed size. A total of 22 candidate genes were enriched in these pathways related to seed size. We infer that these genes may play a key role in the development of cotton seed size and shape.

Based on the functional annotation of orthologs in *Arabidopsis* spp. of 22 candidate genes, 10 candidate genes within the stable QTLs were further identified that may be involved in cotton seed development (Supplementary Table 8). We analyzed the SNP variation of these 10 candidate genes between the two parents, and it contained a total of 1,334 effective SNPs (including intergenic regions). According to the annotations, non-synonymous mutations (9), start gained (1), synonymous variant (7), stop gained (2), splice acceptor variant, and intron variant (1) exist in eight candidate genes, which may affect the biological function of these genes (Table 4).

**TABLE 4:** SNPs in candidate genes between the two parents.

Gene	Position (bp; HAU)	CRI36 base	Hai7124 Base	Mutation type of gene	Effect of SNP	Annotation
*GH_D03G0980*	33,195,805	A	T	5 Prime UTR premature start codon gain variant	LOW	Probable starch synthase 4, chloroplastic/amyloplastic
	33,196,654	T	C	Synonymous variant	LOW	
*GH D03G1091*	36,671,507	C	T	Missense variant	MODERATE	Transcription factor PIF7
*GH D03G1237*	40,735,716	C	A	Missense variant	MODERATE	Endoglucanase 8
	40,742,329	T	C	Missense variant	MODERATE	
	40,738,388	C	A	Synonymous variant	LOW	
*GH D03G1448*	45,225,097	C	T	Stop gained	HIGH	Heat shock cognate 70 kDa protein
	45,224,947	G	C	Missense variant	MODERATE	
	45,223,335	C	T	Synonymous variant	LOW	
*GH D03G1453*	45,306,561	G	A	Stop gained	HIGH	Heat shock cognate 70 kDa protein
	45,307,072	C	T	Splice acceptor variant and intron variant	HIGH	
	45,306,618	T	C	Missense variant	MODERATE	
	45,306,849	T	C	Missense variant	MODERATE	
	45,307,043	C	A	Missense variant	MODERATE	
	45,306,838	T	C	Synonymous variant	LOW	
*GH D03G1458*	45,427,060	C	T	Synonymous variant	LOW	Ubiquitin-conjugating enzyme E2 7
*GH D03G1466*	45,532,073	G	A	Missense variant	MODERATE	THO complex subunit 4A
	45,533,079	A	C	Missense variant	MODERATE	
	45,533,104	A	T	Synonymous variant	LOW	
*GH D12G2619*	59,308,273	T	C	Synonymous variant	LOW	Transcription factor MYC4

**FIGURE 3:**
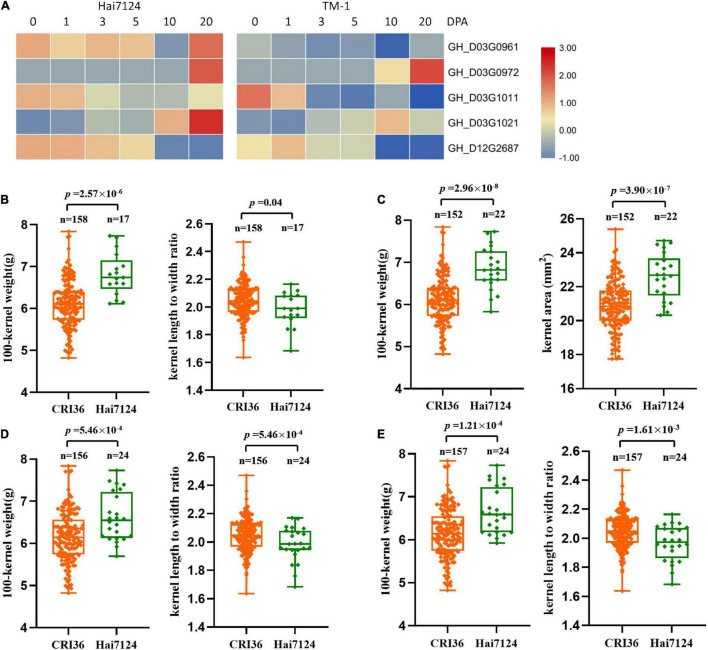
The expression levels and genotypic evaluation of five candidate genes in ovules of each development stage (0, 1, 3, 5, 10, and 20 days post anthesis) of *G. hirsutum* TM-1 and *G. barbadense* Hai7124. **(A)** The expression levels of five candidate genes in ovules of each development stage (0, 1, 3, 5, 10, and 20 days post anthesis) of *G. hirsutum* TM-1 and *G. barbadense* Hai7124. **(B–E)** The distribution and means of seed size and shape traits in the BIL population based on SNP alleles from two parents for GH_D03G0980, GH_D03G1091, GH_D03G1458, and GH_D03G1466, respectively.

Furthermore, we analyzed the expression levels of these eight candidate genes in the ovules of TM-1 (as a proxy to the Upland cotton parent, CRI36) and Hai7124 using previously published RNA-seq data (Hu et al., 2019). Among them, three genes on chromosome D03, *GH_D03G1237*, *GH_D03G1448*, and *GH_D03G1453*, were not expressed during the developmental stages of cottonseed ovules (Supplementary Table 9), and were therefore not further analyzed. Among the remaining five genes, four were on the D03 chromosome including *GH_D03G0980* encoding probable starch synthase 4 (within the region of QTLs- *qKLW-D03-1* and *qKR-D03-1*) and *GH_D03G1091* encoding transcription factor PIF7 (with the region of QTLs- *qHKW-D03-1*, *qKA-D03-1*, *qKG-D03-1*, and *qKD-D03-1*) in a close proximity, and *GH_D03G1458* encoding ubiquitin-conjugating enzyme E27 (within the region of QTLs- *qKLW-D03-1* and *qKR-D03-1*) and *GH_D03G1466* encoding THO complex subunit 4A (within the region of QTLs- *qKLW-D03-1* and *qKR-D03-1*) in a close proximity, and *GH_D12G2619* encoding transcription factor MYC4 (within the region of QTLs- *qKLW-D12-1* and *qKR-D12-1*) on D12. The expression levels of these genes were different during different ovule developmental stages between the two parents (Figure 3A). However, because developing ovules were not harvested for RNA extraction from representative BILs with differing seed physical traits, a comparative quantitative RT-PCR analysis was not performed in this study.

To further determine the effective allelic variation of the candidate genes, we analyzed SNPs of these candidate genes and their contributions to variation in seed size and shape traits. The results showed that SNPs in four candidate genes were significantly associated with changes in seed size and shape (Figures 3B–E). The possible roles of these candidate genes in relation to cottonseed size and shape will be discussed in Discussion.

## Discussion

Crop germplasm, including crop varieties, strains, types, wild species, and relatives, is the source of genes for genetically improving crops (Wang et al., 2005). Cotton, like other crops, has heterosis to varying degrees between species and varieties. Using cotton heterosis is an effective way to increase cotton yield (Xing et al., 2007). Since *G. barbadense* and Upland cotton belong to two different species under the genus *Gossypium*, different genetic loci are involved in seed development. The interspecific hybrids exhibit heterosis, which is reflected in many aspects, such as fiber quality and yield (Zhang et al., 1994, 2014; Wang and Zhe, 2013; Lu et al., 2017). The phenotypic data of all size and shape-related traits between BILs of *G. barbadense* Hai7124 and *G. hirsutum* CRI36 showed rich variation. For example, the minimum value of HKW is 3.63 and the maximum is 9.36, which has obvious transgressive segregation, even though there were no significant differences between the two parents. QTLs mapped by BILs will be the choice for MAS to improve the quality of cotton seeds by transferring favorable alleles to cotton.

In this study, we detected 49 QTLs for cotton seed size and shape-related traits that were distributed in seven QTL clusters, representing one of the first such a comprehensive study in cotton. Nine QTLs were stably detected in multiple environments and were located on chromosomes D03 and D12. Previously, a QTL for plant height (Ma et al., 2019) and a QTL for micronaire (Pei et al., 2021) were detected in this BIL population. Interestingly, the physical interval of the QTL mapping of the seed size and shape overlapped with the QTLs for these two traits. There were also other QTL studies on seed index, with QTLs mapped on the D03 chromosome (Shang et al., 2016; Liu et al., 2018). In a previous QTL mapping study for the four traits (HSW, HKW, TKL, TKW) of cottonseed (Wang et al., 2019), QTLs for the three traits (HSW, HKW, and TKW) were also detected on the D03 chromosome. In addition to two QTL clusters on D03 for the cotton physical traits, our current study detected two new QTLs- *qKLW-D12-1* and *qKR-D12-1* for cotton seed size and shape. These common QTLs and new stable QTLs will be the first choice for MAS to improve cottonseed quality by transferring favorable alleles to cotton cultivars.

Among the 641 genes within nine stable QTLs, we further identified five candidate genes for their possible involvement of regulating seed size and shape based on differential gene expression and sequence variation. The exact roles of these five genes in relation to cottonseed size and shape are currently unknown and should be further studied. The following discussion was solely based on relevant studies in other plants.

*GH_D03G0980* encodes starch synthase 4. Starch synthase 4 (*SS4*) is required for proper starch granule initiation in *Arabidopsis thaliana*; and *ss4* mutants grow poorly even under long-day conditions (Ragel et al., 2013). In rice, four starch synthase I (SSI)-deficient mutant lines did not alter seed morphology (Fujita et al., 2006). Fujita et al. (2011) further showed that rice endosperm requires the presence of either SS I or IIIa for starch biosynthesis, whose mutations led to reduced dehulled seed weight. In wheat, all three SSII genes on A, B and D subgenomes had to be missing or inactive for a change in seed weight and other traits (Konik-Rose et al., 2007). Therefore, seed weight may be affected by SS.

*GH_D03G1091* encodes the transcription factor PIF7, which is a basic helix-loop-helix (bHLH)-type transcription factor. *GH_D12G2619* encodes another bHLH transcription factor *MYC4*, which is homologous to *AT4G00870* in Arabidopsis. This bHLH transcription factor family in plants is widely involved in biological processes, including the response to hormone signals (Friedrichsen et al., 2002; Yin et al., 2005), and flower and fruit development (Rajani and Sundaresan, 2001; Liljegren et al., 2004; Szecsi et al., 2006). It was found in *Arabidopsis* that the bHLH subgroup IIID transcription factors (*bHLH 3*, *bHLH 13*, *bHLH 14*, and *bHLH 17*) have a negative regulatory effect on the jasmonate (JA) response, and can act as a transcription inhibitor to coordinate the JA response, thereby regulating the defense and development of plants (Song et al., 2013). bHLH transcription factors may be also involved in determining seed size and shape. In rice, two bHLH proteins- POSITIVE REGULATOR OF GRAIN LENGTH 1 (PGL1) and its antagonistic partner ANTAGONIST OF PGL1 (APG) were involved in determining rice grain length by controlling cell length in the lemma/palea. Heang and Sassa (2012) showed that overexpression of PGL1 and silencing of APG each increased grain length and weight in transgenic rice, suggesting that APG was a negative regulator whose function was inhibited by PGL1. Other transcription factors can also affect seed size and shape. For example, most recently, Sun et al. (2021) showed that three SNPs related to *ZmBES1/BZR1-5* were significantly correlated with kernel width and four SNPs in the gene were related to 100-kernel weight. They further confirmed that transgenic Arabidopsis and rice with *ZmBES1/BZR1-5* displayed significantly increased seed size and weight, while Mu transposon insertion and EMS maize mutants in the gene possessed smaller kernels.

*GH_D03G1458* encodes the ubiquitin-conjugating enzyme E2 7. The ubiquitin-26S proteasome pathway (*UPP*) is a crucial regulatory mechanism for selective protein degradation in a wide variety of plant developmental processes. Ubiquitin-binding (*UBC*) E2 enzyme, an important part of it, also plays a vital role in plant growth and development (Wang, 2010; Gao et al., 2017). Xu (2014) showed that the null UBC 22 mutants produced larger plants and larger and heavier seeds that stored a higher amount of protein and fatty acids in Arabidopsis. However, Mao et al. (2020) showed that overexpression of a soybean UBC gene (*GmUBC1*) in Arabidopsis significantly increased the 1,000-grain weight and total amino acid content. Similar to genes for *bHLH* transcription factors, different gene family members may have an opposite effect on seed weight and shape.

*GH_D03G1466* encodes THO complex subunit 4A. THO is a multi-protein complex promoting coupling between transcription and mRNA processing. It is demonstrated the THO complex is involved in regulating female germline specification and disease resistance in Arabidopsis (Pan et al., 2012; Su et al., 2017). The destruction of *ALY1*, *ALY2*, *ALY3*, *and ALY4* (orthologs of genes involved in the THO complex) in *Arabidopsis* caused nutritional and reproductive defects, including severe growth slowdowns, changes in flower morphology, and abnormal ovules and female gametophytes, resulting in reduced seed yield (Pfaff et al., 2018). However, the role of the complex in relation to seed size and shape is current unknown.

In cotton, the roles of those five candidate genes in relation to seed physical traits are not understood. However, we showed that these genes may be target genes for the genetic improvement of cotton seed size and shape. Among them, SNPs in four candidate genes were significantly associated with changes in seed size and shape traits such as HKW and KLW. The results provided important alleles for molecular breeding to improve cotton physical traits.

## Conclusion

In summary, 49 QTLs for eight seed size and shape-related traits were identified by QTL mapping using an interspecific BIL population derived from *G. barbadense* Hai7124 and *G. hirsutum* CRI36 as the recurrent parent. Nine stable QTLs and 641 putative genes were identified within these QTL intervals. After further analysis, five genes encoding enzymes and transcription factors were identified as possible candidate genes that may be associated with cotton seed size and shape for further studies. These results represent the first study on the genetic basis for most seed physical traits in cotton. Their relationships with lint percentage and yield and fiber quality should be studied, which will facilitate breeding for high-quality and high-yield cotton.

## Data Availability Statement

The datasets presented in this study can be found in online repositories. The names of the repository/repositories and accession number(s) can be found in the article/Supplementary Material.

## Author Contributions

LWu analyzed, summed all the data, and wrote the manuscript. BJ performed SNP analysis and wrote the introduction part of the manuscript. WP, JM, JS, SY, and MW conducted instrument debugging, managed, and collected phenotypic data. LWa participated in the discussion of the manuscript. YX, LH, and PF participated in the analysis of SNP markers. JY and JZ guided the experiment and manuscript revision. All authors read and approved the final manuscript.

## Conflict of Interest

The authors declare that the research was conducted in the absence of any commercial or financial relationships that could be construed as a potential conflict of interest.

## Publisher’s Note

All claims expressed in this article are solely those of the authors and do not necessarily represent those of their affiliated organizations, or those of the publisher, the editors and the reviewers. Any product that may be evaluated in this article, or claim that may be made by its manufacturer, is not guaranteed or endorsed by the publisher.

## Footnotes

1
https://cottonfgd.org/

2
www.omicshare.com/tools

3
https://bigd.big.ac.cn/bioproject/

## Supplementary Material

Supplementary Figure 1 QTLs for seed size and shape-related traits under five environments and BLUP. The lines on the right side of the linkage groups indicate the QTL likelihood intervals. Map distances in centiMorgans are indicated on the left side of the linkage groups.
